# Flexible Ureterorenoscopy versus Extracorporeal Shock Wave Lithotripsy for the treatment of upper/middle calyx kidney stones of 10–20 mm: a retrospective analysis of 174 patients

**DOI:** 10.1186/2193-1801-3-557

**Published:** 2014-09-24

**Authors:** Kursat Cecen, Mert Ali Karadag, Aslan Demir, Murat Bagcioglu, Ramazan Kocaaslan, Mustafa Sofikerim

**Affiliations:** 1 Department of Urology, Kafkas University Faculty of Medicine, Kars, Turkey; 2 Department of Urology, Acibadem University Faculty of Medicine, Kayseri, Turkey

**Keywords:** Flexible ureterorenoscopy, Shock wave lithotripsy, Upper calyx stones, Mid calyx stones

## Abstract

To compare the outcomes of flexible ureterorenoscopy (F-URS) with extracorporeal shock wave lithotripsy (ESWL) for the treatment of upper or mid calyx kidney stones of 10 to 20 mm.

A total of 174 patients with radioopaque solitary upper or mid calyx stones who underwent ESWL or F-URS with holmium:YAG laser were enrolled in this study. Each group treated with ESWL and F-URS for upper or mid calyx kidney stones were retrospectively compared in terms of retreatment and stone free rates, and complications.

87% (n = 94) of patients who underwent ESWL therapy was stone free at the end of 3rd month. This rate was 92% (n = 61) for patients of F-URS group (p = 0.270 p > 0.05). Retreatment was required in 12.9% of patients (n = 14) who underwent ESWL and these patients were referred to F-URS procedure after 3rd month radiologic investigations. The retreatment rate of cases who were operated with F-URS was 7.5% (n = 5) (p = 0.270 p > 0.05). Ureteral perforation (Clavien grade 3B) was occured in 3 patients (4.5%) who underwent F-URS. Fever (Clavien grade 1) was noted in 7 and 5 patients from ESWL and F-URS group, respectively (6.4% vs 7.5%) (p = 0.78 p > 0.05).

F-URS and ESWL have similar outcomes for the treatment of upper or mid calyx renal stones of 10–20 mm. ESWL has the superiority of minimal invasiveness and avoiding of general anethesia. F-URS should be kept as the second teratment alternative for patients with upper or mid caliceal stones of 10–20 mm and reserved for cases with failure in ESWL.

## Introduction

The European Association of Urology (EAU), updates and publishes guidelines for urolithiasis and treatment algorithms every year. EAU 2014 guidelines state that stones between 10–20 mm in every locations should be treated with extracorporeal shock wave lithotripsy (ESWL) or endourologic interventions (Türk et al. 2014). With the miniaturization and advancements in the designs of ureterorenoscopes, stone disintegration systems and endourologic techniques, most of the kidney stones and large proximal ureteral stones can be managed by flexible ureterorenoscopy (F-URS) nowadays (Bas et al. 2013; Ching-Fang et al. 2004). Especially introduction of holmium:YAG laser into the market and worlwide accepted use of this laser during URS makes the stone clearence rates better even for the renal stones up to 20 mm (El-Nahas et al. 2013).

In this study, we aimed to compare the outcomes of F-URS with ESWL for the treatment of radioopaque solitary upper or mid calyx kidney stones of 10 to 20 mm.

## Materials and methods

The medical files of 563 patients who underwent ESWL or F-URS for kidney stones in Kars State Hospital, Kafkas University Faculty of Medicine and Acibadem Kayseri Hospital between april 2008 and december 2012 were reviewed and database of the study was formed. ESWL was performed in 421 patients and 142 underwent F-URS. The data of the eligible patients having solitary upper or mid calyx stones with 10–20 mm were matched according to the stone size, age, gender and body mass index (BMI). According to the data searched, a total of 174 patients with radioopaque solitary upper or mid calyx stones who underwent ESWL or flexible ureterorenoscopic lithotripsy with holmium:YAG laser were enrolled in this study. Review of the complete medical records of the patients for our study was approved by local ethics committee of Kafkas University Faculty of Medicine and performed in accordance with the Helsinki Declaration of the World Medical Association. All of the patients signed and understood informed consent forms about applications.

Inclusion criteria of this study was patients who were treated for solitary radioopaque upper or mid calyx stones with ESWL or F-URS and who had post-procedure 3rd month radiological investigations for assessment of stone free status in the medical records. Patients with kidney stones who were previously operated, cases with ureteropelvic junction obstruction, solitary kidneys or multiple stones and the patients under 18 years old were excluded from the study. The imaging investigations were plain X ray of the kidneys, ureter and bladder (KUB), urinary ultrasonography and non contrast computed tomography of the abdomen when required.

Stone free status was assessed with plain X ray of the kidneys, ureter and bladder (KUB), urinary ultrasonography (USG) or non contrast computed tomography of the abdomen at post-procedure 3rd month in 54, 58 and 62 patients, respectively. Stone free status was accepted as complete stone clearance or clinically insignificant residual fragments (CIRF) (<2 mm) observed at 3rd month radiologic investigations. Retreatment was defined as F-URS requirement for patients who failed at the end of 3 sessions of ESWL therapy and secondary F-URS need for patients having inadequate disintegration observed at the 3rd month radiologic investigation.who were treated with F-URS previously.

### F-URS procedure

F-URS procedures were performed in Acibadem Kayseri Hospital. A 7.5 F flexible ureterorenoscope (Flex-X2, Karl Storz, Tuttlingen, Germany) was used in all operations. After introducing a safety guide wire to the ureter, a 9.5/11.5 F access sheath (Cook Urological, Spencer, USA) was inserted over the guide wire with the aid of C arm fluoroscopy. Then, the flexible ureterorenoscope passed through the access sheath under direct vision. After reaching the kidney stones, disintegration was completed by using 20 W holmium:YAG laser (Lumenis, Santa Clara, USA). A 200-μm laser fibre with an energy output of 0.8-1.5 J at 8–12 Hz was preferred; but the joule and hertz of energy could be changed during the operation according to the stone hardness and efficacy of lithotripsy. The main goal was to disintegrate the stones until the fragments were smaller than 2 mm under direct vision. In case of failure in F-URS, a second look procedure was planned. If there was a serious stenosis or tortuosity of the ureter and acess sheat could not be inserted, the access sheat was removed and we directly advanced the flexible ureterorenoscope over the guide wire under C-arm fluoroscopy.

### ESWL procedure

All of the procedures were performed by an experienced urologist on ESWL therapy and a technician with Siemens Lithostar machine in Kars State and Kafkas University Faculty of Medicine Hospital. An intramusculer non steroidal anti-inflamatory drug was administered before the sessions. We did not prefer any sedoanalgesia or general anesthesia. Stone location was identified with the aid of fluoroscopy. A maximum of 3000 shocks were applied at 80 shocks per minute during each session or until complete disintegration of the stones were observed. All of the cases were evaluated with KUB and urinary USG after 1 week and if the stone fragmentation was inadequate, another session was planned. The maximum number of sessions allowed per patient was 3 and if failure occured at the end of 3 sessions, patient was referred for F-URS procedure.

### Statistical analysis

The data were analyzed by using SPSS for windows version 16.oo. Means, medians and standard deviations were used for descriptive statistics. The characteristics of the 2 operative groups were compared with each other. The characteristics with normal and non normal distributions were compared by using Student T and Mann Whitney tests, respectively. A *p* value of <0.05 was considered significant.

Each group treated with ESWL and F-URS for upper or mid calyx kidney stones were compared in terms of retreatment rates, stone free status at 3rd month and complications. Modified Clavien grading system was preferred for classification of complications (Dindo et al. 2004).

## Results

There was not any statistical difference between two groups in terms of age, gender, stone size, BMI, retreatment and stone free rates (Table 1). Retreatment was required in 12.9% of patients (n = 14) who underwent ESWL and these patients were referred to F-URS procedure after 3rd month radiologic investigations. Of these patients, 9 had stones resistant to ESWL therapy and 5 were unable to pass the stone fragments. All of them were stone free after F-URS. The retreatment rate of cases who were operated with F-URS was 7.5% (n = 5) (Table 1). We performed a second look F-URS to these patients and all of them were stone free after 2nd intervention. In 12 patients from F-URS group, access sheath could not be passed over guide wire due to stenosis or tortuosity of the ureters. We removed the access sheaths of these cases and directly advanced the flexible ureterorenoscope over the guide wire under direct vision with the aid of fluoroscopy. We successfully reached to the stones of these patients and disintegrated the stones.

**Table 1:** **Patient demographics and comparison of outcomes between 2 groups**

Variables	ESWL (n = 108)	F-URS (n = 66)	P
Mean patient age	39.47 ± 9.80	40.74 ± 1.12	0.434
Sex			0.636
Female	51	33	
Male	57	33	
Stone size(mm)	12.89 ± 2.54	13.31 ± 2.63	0.299
BMI	26.9 ± 0.72	27.06 ± 0.93	0.326
Success rate	87.04% (n = 94)	92.42% (n = 61)	0.270
Retreatment	12.9% (n = 14)	7.5% (n = 5)	0.270
Clavien Grade			
1	n = 7	n = 5	0.783
2	n = 11	n = 7	0.930
3			
A			
B	0	n = 3	0.026
4	0	0	
5	0	0	

Complications were classified according to modified Clavien Grading system (Dindo et al. 2004). No major intraoperative or post-procedure complications (Clavien grade 4–5) like ureteral avulsion or septicaemia were encountered. Ureteral perforation (Clavien grade 3B) was occured in 3 patients (4.5%) who underwent F-URS. All of them were observed in the proximal ureter and the reason was forced advancement of flexible ureterorenoscope while attempting to pass the tortuosity of the ureters. The perforations were minimal and managed with insertion of the double j ureteral stents. They were reoperated 14 days later. Fever (Clavien grade 1) was noted in 7 and 5 patients from ESWL and F-URS group, respectively (6.4% vs 7.5%, p > 0.05). They were treated with antipyretics and resolved spontaneously approximately 36 hours later. Urinary tract infections (Clavien grade 2) were noted in 11 cases (10.1%) from ESWL group; besides this only 7 patients (10.6%) who underwent F-URS suffered from urinary infection (p > 0.05) (Table 1). They were managed with nitrofurantoin 500 miligram (3 times daily) successfully and free of sypmtoms approximately 48 hours after onset of administration.

## Discussion

The management of upper urinary tract stones is a complex issue and may vary according to the stone size, location, factors related to the patient and armamentarium of the department. Treatment alternative consists of a huge spectrum including modalities like F-URS, ESWL, percutaneous nephrolithotomy (PCNL), laparoscopy, mini-PCNL and open surgery. There are some studies in the literature dealing with the relationship between the size of the stone and success rate of the treatement modality. Success rates of F-URS and ESWL are affected from the size of the stone; besides this PCNL is hardly affected (Argyropoulos and Tolley 2010; Srisubat et al. 2009). ESWL obtains excellent success rates for renal stones up to 20 mm, except for the lower pole’s (Sahinkanat et al. 2008; Preminger 2006). Success rate of ESWL therapy may reach 90% for patients having lower pole stones, if the size of the stones is < 1 cm (Sener et al. 2013). EAU 2014 guidelines advices ESWL as the first line treatment alternative for stones < 2 cm located in the renal pelvis, upper or middle calices (Türk et al. 2014). Stones > 2 cm should be treated with PCNL due to the reasons that ESWL choice for these stones would be less successful, require auxiliary procedures, be challenging and cause complications like colic or steinstrasse (Türk et al. 2014; Pearle et al. 2005). F-URS is not advised for patients having renal,upper or middle pole stones > 15 mm due to low success rates and need for staged applications (Türk et al. 2014). The patients having renal stones >20 mm were not included in this study, because we preferred PCNL for these stones.

The success rate of ESWL was reported to be between 60-90% in the literature (Abe et al. 2005; Egilmez et al. 2007). We achieved an overall stone free rate of 87% in patients who underwent ESWL and it was similar with the results in the literature. A recent study dealed with the outcomes between retrograde intrarenal surgery, shockwave lithotripsy, and percutaneous nephrolithotomy for treatment of medium-sized radiolucent renal stones (Resorlu et al. 2012a). ESWL group consisted of 251 patients. Ninety nine of them had pelvis, 48 had upper/middle pole, 72 had lower pole and 32 had multicaliceal stones. By using an electromagnetic or electrohydraulic lithotripter, they targeted the stones with ultrasonography and reveled an overall stone free rate of 66.5%. We achieved a stone free rate of 87% in our patients who underwent ESWL. This difference may be attributable to the difference in patient selection of 2 studies. The stones were radioopaque, solitary and located either in the upple or middle calyx in our study; on the other hand the patient selection of the other study was more difficult than ours. They included the cases having radioluscent stones with different locations like lower pole or multiple caliceal stones that diminished their stone free rate.

Recent developments in the market about miniaturization of semirigid and flexible ureterorenoscope and holmium:YAG laser in URS applications attracted the attentions’ of the urologists and markedly improved the success rates of management of kidney stones. In a recent study, 47 patients with a mean stone size of 14.8 ± 2.3 mm were treated with F-URS by using Flex-X2® (Karl Storz, Tuttlingen, Germany) (Bas et al. 2013). The investigators revealed a stone free rate of 91.4% at the end of 1st month. The main reason of failure was migration of the kidney stones into the lower pole in 3 patients. Even the inclusion criteria for our study was patients having isolated solitary upper or middle calyx stones, our success rate for patients who underwent F-URS was 92% and it was similar with the success rate of mentioned study. Success rate of F-URS in patients having pyelocaliceal stones were investigated in a study (Geavlete et al. 2006a). The authors concluded that complete stone clearance or CIRF smaller than 3 mm was obtained in 57% for pyelocaliceal calculi. The stone free rate of this study was lower than ours due to the reasons that some stones were larger 20 mm and inclusion of multiple calyceal stones; besides this, the stones in our study were solitary and smaller than 20 mm.

Despite the fact that, we did not investigate ESWL and F-URS in terms of cost; the major disadvantage of F-URS over ESWL seems to be high cost and requirement of expensible repairments especially in the learning curve. Addition of special instruments developed for flexible procedures like laser fibres and tipless nitinol baskets also increase the bill of this procedure. In 2000, 2 stuides investigated the durability and proper functionality of flexible ureterorenoscopes. One study reported repairement of flexible instrument after 12 cases; on the other hand the other reported same issue after 21 cases (Afane et al. 2000; Hollenbeck et al. 2000). The main reason of the repair was reported to be the malfunction of the active deflection mechanism (Monga et al. 2006). Some maneuvers for prevention of prolonged deflection have been proposed like displacing the lower pole stone into the easily accessible calyx like upper poles (Schuster et al. 2002). We broke and repaired the flexible ureterorenoscope 2 times during the operations of 66 cases. Our study’s advantage was including the cases having upper or mid calyx stones instead of lower calyx; by this way we avoided of excessive deflection of the flexible ureterorenoscopy.

The effect of stone related factors and pelvi-calyceal anatomy on success rate of F-URS was evaluated in a recent study (Resorlu et al. 2012b). The authors revealed that lower pole infundibulopelvic angle (IPA) and stone size were the most important factors affecting success rate after F-URS. A high success rate as 91% could be achieved in the presence of IPA > 45°. We obtained a stone free rate of 92% and this was mainly due to not having patients with lower pole stones and not facing with such an important factor like IPA.

The most important and serious complications of ureteroscopic lithotripsy are ureteral avulsion and perforation. One of the large series dealing with the complications of ureteroscopy was reported in 2006 (Geavlete et al. 2006b). The authors reported serious complications as fever or sepsis (1.13%), ureteral avulsion (0.1%), and ureteral strictures (0.1%). The minor complications were mucosal injury (1.5%), double j stent disposition (0.77%) and renal colic (2.2%). We did not encounter Clavien grade 4–5 complications like septicaemia or ureteral avulsion in our study. As Clavien grade 3B complication, ureteral perforation was occured in 3 patients and they were treated conservativelty with the insertion of double j ureteral stent. We think that it was due to surgeon’s forced forward pushing of flexible ureterorenoscope for overcoming angulations, tortuousity of the ureter. All of the perforations were observed in patients who were not inserted access sheath over guide wire due to tortuosity or the serious stenosis of the ureter. In our opinion, if we succeeded at insertion of the access sheaths, we would not encounter perforations in these patients. In ESWL group, no major complications or perirenal, intrarenal or subcapsular hematomas were noted. The comparison between 2 groups in terms of fever and urinary tract infection was statistically insignificant.

There are several limitations of our study. First of all, our study had a retrospective nature and based on a small sample size. Postoperative or post-procedure pain scores, analgesic need and stone composition of the patients which can significantly affect the outcomes of the procedures were not evaluated in our study. Also we did not perform and compare a cost-effectivity analysis for F-URS and ESWL.

F-URS and ESWL have similar stone free and complication rates for the treatment of upper or mid calyx renal stones of 10–20 mm. ESWL has the superiority of minimal invasiveness and avoiding of general anethesia. Depending on these facts, it can be concluded that F-URS should be kept as the second treatment alternative for patients with upper or mid caliceal stones of 10–20 mm and reserved for cases with failure in ESWL. The high cost and expensive repairement of the flexible ureterorenoscope should also be kept in mind in the management of these patients.
