# Erratum to: Flexible Ureterorenoscopy versus Extracorporeal Shock Wave Lithotripsy for the treatment of upper/middle calyx kidney stones of 10–20 mm: a retrospective analysis of 174 patients

**DOI:** 10.1186/s40064-016-2112-x

**Published:** 2016-04-19

**Authors:** Kursat Cecen, Mert Ali Karadag, Aslan Demir, Murat Bagcioglu, Ramazan Kocaaslan

**Affiliations:** Department of Urology, Kafkas University Faculty of Medicine, Kars, Turkey

## Erratum to: SpringerPlus (2014) 3:557 DOI 10.1186/2193-1801-3-557

After publication of this article (Cecen et al. 2014) we received a request from Dr. Mustafa Sofikerim to have his name removed from the author list as he felt he did not fully meet the authorship criteria. The correct author list is above.
